# Efficacy of Massage Therapy on Pain and Dysfunction in Patients with Neck Pain: A Systematic Review and Meta-Analysis

**DOI:** 10.1155/2014/204360

**Published:** 2014-02-20

**Authors:** Yong Hong Cheng, Gui Cheng Huang

**Affiliations:** ^1^Nanjing University of Traditional Chinese Medicine, Nanjing, Jiangsu 210023, China; ^2^Department of Spinal Surgery of the First People's Hospital of Hefei, 390 Huaihe Road, Hefei 230061, China

## Abstract

*Objective*. To systematically evaluate the evidence of whether massage therapy (MT) is effective for neck pain. *Methods*. Randomized controlled trials (RCTs) were identified through searches of 5 English and Chinese databases (to December 2012). The search terms included neck pain, neck disorders, cervical vertebrae, massage, manual therapy, Tuina, and random. In addition, we performed hand searches at the library of Nanjing University of Traditional Chinese Medicine. Two reviewers independently abstracted data and assessed the methodological quality of RCTs by PEDro scale. And the meta-analyses of improvements on pain and neck-related function were conducted. *Results*. Fifteen RCTs met inclusion criteria. The meta-analysis showed that MT experienced better immediate effects on pain relief compared with inactive therapies (*n* = 153; standardised mean difference (SMD), 1.30; 95% confidence interval (CI), 0.09 to 2.50; *P* = 0.03) and traditional Chinese medicine (*n* = 125; SMD, 0.73; 95% CI 0.13 to 1.33; *P* = 0.02). There was no valid evidence of MT on improving dysfunction. With regard to follow-up effects, there was not enough evidence of MT for neck pain. *Conclusions*. This systematic review found moderate evidence of MT on improving pain in patients with neck pain compared with inactive therapies and limited evidence compared with traditional Chinese medicine. There were no valid lines of evidence of MT on improving dysfunction. High quality RCTs are urgently needed to confirm these results and continue to compare MT with other active therapies for neck pain.

## 1. Introduction

Neck pain is a very common condition. It has one-month prevalence between 15.4% and 45.3% and 12-month prevalence between 12.1% and 71.5% in adults [1]. Despite its high prevalence, neck pain frequently becomes chronic and affects 10% of males and 17% of females [2].Consequently, neck pain has been a source of disability and may require substantial health care resources and treatments [3–6].

Massage therapy (MT), as one of the earliest and most primitive tools for pain, has been widely used for neck pain. It is defined as a therapeutic manipulation using the hands or a mechanical device, in which numerous specific and general techniques are used in sequence, such as effleurage, petrissage, and percussion [7]. There are, however, inconsistent conclusions on effects of MT for neck pain. Some prior reviews maintained that there was inconclusive evidence on effects of MT for neck pain [8–11], but the others suggested that MT had immediate effects for neck pain [12, 13]. In addition, most reviews did not include Chinese randomized controlled trials (RCTs) of MT for neck pain due to language barrier or limited retrieving resources [8, 9, 11, 12]. But Chinese MT, as one of the primitive complementary and alternative treatments, has been employed by most Chinese patients with neck pain, and a mass of studies have been reported [10]. They are important for evaluating the evidence of MT for neck pain.

Therefore, we performed an updated systematic review of all currently available both English and Chinese publications and conducted quantitative meta-analyses of MT on neck pain and its associated dysfunction to determine whether MT is a viable complementary and alternative treatment for neck pain.

## 2. Materials and Methods

The following electronic databases were searched from their inception to December 2012: PubMed, EMBASE, Cochrane Library, China Knowledge Resource Integrated Database (CNKI), and Wan Fang Data. The main search terms were neck pain, neck disorders, cervical vertebrae, massage, manual therapy, Tuina, and random. And we performed hand searches at the library of Nanjing University of Traditional Chinese Medicine. Reference lists of retrieved articles were also screened. No restrictions on publication status were imposed.

### 2.1. Eligibility Criteria

Only the studies that met the following criteria were included: (1) RCTs of MT for neck pain; (2) neck pain was not caused by fractures, tumors, infections, rheumatoid arthritis, and so forth; (3) MT was viewed as an independent therapeutic intervention for neck pain, which did not combine with other manual therapies such as spinal manipulation, mobilization, and chiropractic; (4) the control interventions included inactive and active therapies; the inactive therapy controls included sham, placebo, no treatment, standard care, and others (i.e., massage + exercise versus exercise); the active therapy controls may be any active treatment not related to MT; (5) the main outcome measures were pain and neck-related dysfunction; no restrictions were set on the measurement tools used to assess these outcomes, since a large variety of outcome measures were employed in the studies; (6) the language was either English or Chinese.

### 2.2. Data Abstraction

Two reviewers independently extracted data onto predefined criteria in Table 1. We contacted primary authors when relevant information was not reported. Differences were settled by discussion with reference to the original article. For crossover studies, we considered the risk for carryover effects to be prohibitive, so we selected only the first phase of the study. We considered that effects of MT included immediate effects (immediately after treatments: up to one day) and follow-up effects (short-term follow-up: between one day and three months, intermediate-term follow-up: between three months and one year, and long-term follow-up: one year and beyond).

### 2.3. Methodological Quality Assessment

The methodological quality of RCTs was assessed independently in line with PEDro scale by two reviewers, which is based on the Delphi list and has been reported to have a fair to good reliability for RCTs of the physiotherapy in systematic reviews. And the authors compared the results and discussed difference according to the PEDro operational definitions until agreement was reached. The PEDro score ranged from 0 to 10, and a higher score represents a better methodological quality. A cut point of 6 was used to indicate high quality studies as it has been reported to be sufficient to determine high quality versus low quality in previous studies [14, 15]. If additional clarification was necessary, we contacted primary authors.

### 2.4. Data Synthesis and Analysis

The detailed subgroup meta-analyses were performed based on different control therapies. Each subgroup should include at least 2 RCTs. Standardised mean difference (SMD) was used in meta-analyses because the eligible studies assessed the outcome based on different scales (e.g., VAS 0–10 and VAS 0–100). And the SMD and 95% confidence intervals (CI) were calculated in the meta-analyses. We used the more conservative random effects model to account for the expected heterogeneity. The *I* ^2^ was used to assess statistical heterogeneity. The reviewers determined that heterogeneity was high when the *I* ^2^ was above 75% [16]. The Cochrane Collaboration software (Review Manager Version 5.0 for Windows; Copenhagen: The Nordic Cochrane Centre) was used for the meta-analyses.

## 3. Results

**Figure 1:**
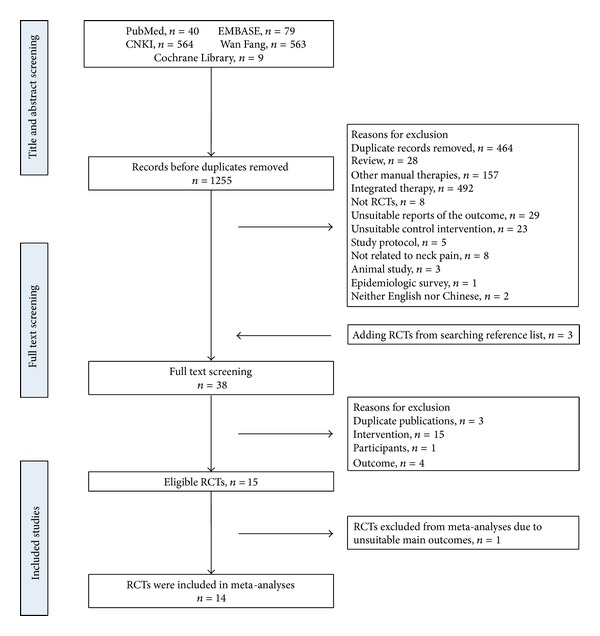
Study selection process. RCTs: randomized controlled trials.

We identified 1255 records from English and Chinese databases. After the initial titles and abstracts screening, we excluded 1220 because of a large number of duplicate records and because some reports failed to meet the inclusion criteria. We retrieved and reviewed 38 full articles including 3 studies from the reference lists of related reviews. 15 RCTs were eligible [17–31]. Of all the excluded studies, the trials were excluded due to duplicate publications (*n* = 3), interventions (*n* = 15), participants (*n* = 1), and outcomes (*n* = 4) in Table 2. And one RCT was excluded from meta-analyses for its unsuitable main outcomes [22]. The study selection process was summarized in Figure 1.

One study was contacted to request for mean and standard deviation data on primary outcomes [24]. Another trial was contacted to provide details on therapeutic technique and study design [31].

### 3.1. Study Characteristics

Fifteen eligible studies including 1062 subjects with mean age of 41.9 ± 12.4 were, respectively, conducted in Australia, China, Finland, Germany, Poland, Spain, USA, and UK between 2001 and 2012. The disease duration ranged from 1 week to 11.2 years and the study duration 1 day to 10 weeks. The session and time of MT, respectively, were 8.1 ± 5.6 (range 1–18) and 31.1 ± 11.7 minutes (range 20–60 minutes). The follow-up time ranged from 6 to 48 weeks.

MT in the studies included Chinese traditional massage, common Western massage, manual pressure release, strain/counterstrain technique, and myofascial band therapy. The control therapies contained inactive therapies (standard care and sham therapies) and active therapies including acupuncture, traction, physical therapy, exercise, traditional bone setting, traditional Chinese medicine, joint mobilization, and activator trigger point therapy. The characteristics of all studies were summarized in Table 1.

### 3.2. Methodological Quality

The quality scores were presented in Table 3. The quality scores ranged from 5 to 9 points out of a theoretical maximum of 10 points. The most common flaws were lack of blinded therapists (87% of studies) and blinded subjects (80% of studies). Although all studies adopted random assignment of patients, eight trials did not use adequate method of allocation concealment [17–20, 23, 25, 30, 31]. The blinded assessors were not performed in six trials [25, 27–31]. Four studies were lacking of analysis by intention-to-treat because they cancelled the dropout data in the last results [18, 21, 22, 29]. For other items on PEDro scale, the included studies showed higher methodological quality in measure of similarity between groups at baseline, less than 15% dropouts, between-group statistical comparisons, and point measures and variability data.

### 3.3. The Effects of MT on Pain

**Figure 2:**
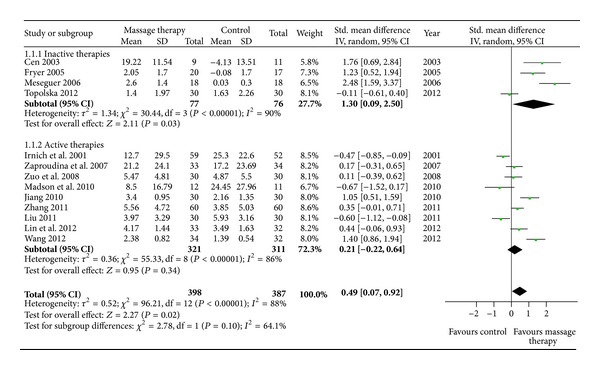
Forest plot of the immediate effect of MT on pain. CI: confidence interval; IV: independent variable; Std.: standard.

Fourteen RCTs examined the immediate effect of MT for neck pain versus inactive therapies or active therapies. Thirteen of them were included in the meta-analysis [17–21, 23, 25–31]. The aggregated results suggested that MT showed better immediate effects on pain relief (*n* = 785; SMD, 0.49; 95% CI 0.07 to 0.92; *P* = 0.02, in Figure 2). But the subgroup meta-analysis suggested that MT only showed superior immediate effects on pain relief compared with inactive therapies (*n* = 153; SMD, 1.30; 95% CI 0.09 to 2.50; *P* = 0.03, in Figure 2).

**Figure 3:**
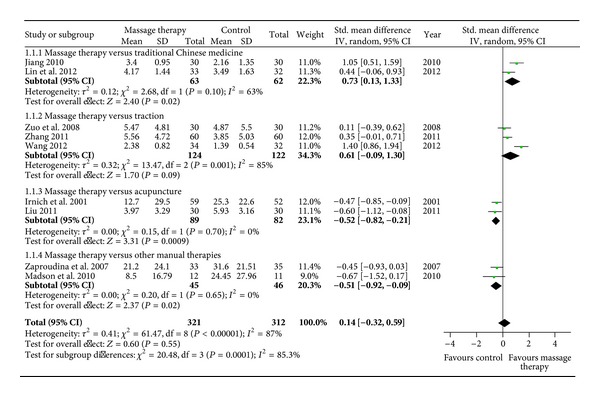
Forest plot of the immediate effect of MT on pain versus different active therapies. CI: confidence interval; IV: independent variable; Std.: standard.

Although MT did not show significant immediate effects on pain relief compared with active therapies (*n* = 632; SMD, 0.21; 95% CI −0.22 to 0.64; *P* = 0.34, in Figure 2), MT showed superior immediate effects on pain relief versus traditional Chinese medicine (*n* = 125; SMD, 0.73; 95% CI 0.13 to 1.33; *P* = 0.02, in Figure 3) in subgroup meta-analyses based on different active therapies. However, MT did not show significant immediate effects on pain relief versus traction (*n* = 246; SMD, 0.61; 95% CI −0.09 to 1.30; *P* = 0.09, in Figure 3). What is more, acupuncture (*n* = 171; SMD, −0.52; 95% CI −0.82 to −0.21; *P* = 0.0009, in Figure 3) and other manual therapies (*n* = 91; SMD, −0.51; 95% CI −0.92 to −0.09; *P* = 0.02, in Figure 3) showed superior immediate effects on pain relief versus MT.

**Figure 4:**
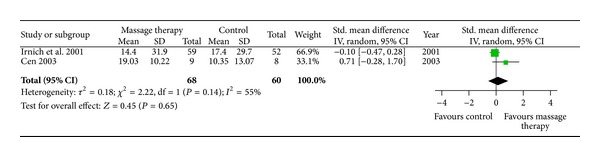
Forest plot of follow-up effects of MT on pain. CI: confidence interval; IV: independent variable; Std.: standard.

With regard to pain relief, two RCTs assessed short-term effects of MT compared with acupuncture after 12 weeks of follow-up (*n* = 111; SMD, −0.10; 95% CI −0.47 to 0.28, in Figure 4) [17] and exercise after 6 weeks of follow-up (*n* = 17; SMD, 0.71; 95% CI −0.28 to 1.70, in Figure 4) [18]. One trial tested the intermediate-term effect of MT versus traditional bone setting (VAS mean improvements, 16.53 versus 23.97) and physical therapy (VAS mean improvements, 16.53 versus 13.54) after 48 weeks of follow-up [21]. The other trial did not report detailed results [28].

### 3.4. The Effects of MT on Dysfunction

**Figure 5:**
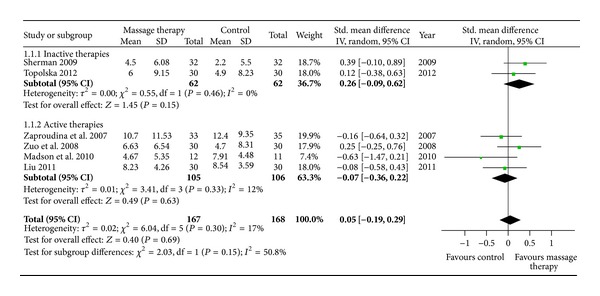
Forest plot of the immediate effect of MT on dysfunction. CI: confidence interval; IV: independent variable; Std.: standard.

Six RCTs examined the immediate effect of MT on dysfunction by neck disability index (NDI) versus inactive therapies [24, 31] or active therapies [21, 23, 26, 27]. All of them were included in the meta-analysis. The aggregated results suggested that MT did not show significant immediate effects on dysfunction compared with inactive therapies (*n* = 124; SMD, 0.26; 95% CI −0.09 to 0.62; *P* = 0.15, in Figure 5) or active therapies (*n* = 211; SMD, −0.07; 95% CI −0.36 to 0.22; *P* = 0.63, in Figure 5).

**Figure 6:**
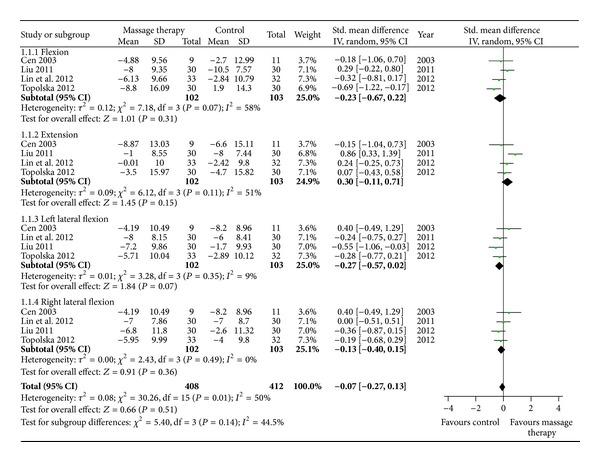
Forest plot of the immediate effect of MT on range of motion. CI: confidence interval; IV: independent variable; Std.: standard.

Four RCTs assessed the immediate effect of MT on range of motion of the neck compared with exercise (or standard care) [18], acupuncture [27], traditional Chinese medicine [29], and physical therapy [31]. MT did not show superior effects in range of flexion (*n* = 205; SMD, −0.23; 95% CI −0.67 to 0.22; *P* = 0.31, in Figure 6), extension (*n* = 205; SMD, 0.30; 95% CI −0.11 to 0.71; *P* = 0.15, in Figure 6), left lateral flexion (*n* = 205; SMD, −0.27; 95% CI −0.57 to 0.02; *P* = 0.07, in Figure 6), or right lateral flexion (*n* = 205; SMD, −0.13; 95% CI −0.40 to 0.15; *P* = 0.36, in Figure 6).

Two trials assessed the follow-up effects of MT on functional improvements by NDI. One study assessed intermediate-term effects of MT compared with traditional bone setting (mean improvements, 4.58 versus 9.46) and physical therapy (mean improvements, 4.58 versus 6.20) after 48 weeks of follow-up [21]. The other tested intermediate-term effects of MT were compared with standard care (mean improvements, 4.7 versus 2.8) after 16 weeks of follow-up [24].

### 3.5. Adverse Events

Only two studies reported side effects. One study reported that 21% of the participants experienced low blood pressure following treatment [17]. The other trial reported that 9 (about 28%) participants had mild adverse experiences including discomfort, pain, soreness, and nausea [24].

## 4. Discussion

The purpose of our systematic review was to evaluate the evidence of MT for neck pain. Our meta-analyses found beneficial evidences of MT for neck pain. Compared with inactive therapies, MT showed moderate evidence for immediate improvement of pain, and compared with traditional Chinese medicine there was limited evidence for immediate improvement of pain due to few eligible studies. However, MT did not show better effects versus other active therapies (including acupuncture, traction, and other manual therapies). And there was no evidence that MT showed superior immediate effects on improving dysfunction in patients with neck pain. On follow-up effects, there was not enough evidence of MT for neck pain.

Our review contained six Chinese RCTs of MT for neck pain. Although MT is widely used for neck pain in China, most of the previous reviews included few Chinese RCTs of MT for neck pain due to limitations of retrieving resources and methodological qualities. In our review, all Chinese RCTs performed eligible random allocation and the quality scores were more than 6 in terms of PEDro scores. They failed to blind the subjects and therapists, but three RCTs [27–29] performed eligible concealed allocation, and one [23] employed blinded assessors. What is more, it is difficult to blind the patients and therapists in MT studies. In general, methodological quality of Chinese RCTs of MT for neck is becoming better.

In our review, there were more detailed subgroup analyses based on inventions of control groups. In order to address the question of what her MT is an effective therapy for neck pain, we analyzed studies comparing MT with inactive therapies including sham therapies and standard care. The result only showed that MT may be more effective than standard care. And we also compared MT with active therapies including acupuncture, traction, traditional Chinese medicine, physical therapy, exercise, and other manual therapies for assessing the question of what her MT is a better therapy for neck pain. The meta-analysis showed that MT has better immediate effects than traditional Chinese medicine, but eligible studies were few. And the treatment process of traditional Chinese medicine is usually longer; 3 to 4 weeks of traditional Chinese medicine may be shorter for neck pain [25, 29]. So we considered that MT did not show better effects than other active therapy. In addition, we also paid attention to dysfunction related neck pain and follow-up effects of MT for neck pain.

### 4.1. Agreements and Disagreements with Other Reviews

The Patel systematic review was the most last review of MT for neck pain, which included fifteen trials (published from 2003 to 2009) with low or very low methodological quality. And it supported the effectiveness of massage for neck pain remained uncertain [8]. Its result concurred with the result of our review, but our review excluded a few studies that Patel had included because they used treatments related to MT in control groups [55–58]. These were limited to evaluating the specific effect of MT. And some studies were not eligible for inclusion criteria of our review [59–62]. Moreover, our systematic review included eight new RCTs [23, 25–31] published from 2008 to 2012. Of notes, our review contained six Chinese RCTs of MT for neck pain [23, 25, 27–30]. And we assessed the effect of MT on neck pain and its associated dysfunction. We also paid attention to the immediate and follow-up effects of MT. So our update provides stronger evidence of MT for neck pain.

Our results differ from systematic reviews [12, 13]. Ottawa panel evidence-based clinical practice guidelines, including five RCTs with high methodological quality (>3) according to the Jadad scale, suggested that MT was effective for relieving immediate posttreatment neck pain symptoms [12]. One suspected reason for this difference is that a mass of new RCTs [20, 21, 23, 25–31] have been published, which were not included in their review. Another possible explanation for the difference is that Jadad scale was replaced by PEDro scale in our review, which is a more detailed method based on the Delphi list and has been reported to have a fair to good reliability for RCTs of the physiotherapy in systematic reviews. In addition, detailed meta-analyses were performed based on more RCTs in our review. Ottawa panel clinical practice guidelines declined to combine the trials because of fewer trials. Moreover, we separately compared MT with inactive therapies and active therapies, and assessed the effect of MT on neck pain and its associated dysfunction in our review. More eligible RCTs, classification of quantitative data synthesis, and detailed assessment of MT on neck pain and its associated dysfunction strengthened our confidence in our systematic review.

### 4.2. Limitations

There are several limitations in our review as follows. (a) Although the predetermined cutoff 6 was exceeded, there were serious flaws in blinding methods of most Chinese RCTs. It is difficult to blind the patients and impossible to blind the therapists, but blinded assessors and concealed allocation must attempt to make up for the lack of blinding. However, some Chinese RCTs did not perform these compensated methods. Thus, these studies could not be considered to be of high quality. (b) Our review may also be affected by dosing parameters of MT such as duration (time of each MT), frequency (sessions of MT per week), and dosage (size of strength). MT commonly combines different techniques (stroking, kneading, percussion, etc.), and each therapist may perform them in different dosing parameters. So the dose-finding studies are warranted to establish a minimally effective dose. (c) The results may be influenced by different outcome measures of pain and dysfunction in eligible RCTs. So the reliable and valid outcome measures is essential to reduce bias, provide precise measures and perform valid data synthesis. (d) There were less eligible trials in some subgroups of meta-analyses because of strict eligibility criteria for considering studies in our review. It may influence combining results, but low eligibility criteria would generate more doubtful results. (e) The majority of trials did not report adverse events, so it was not clear from the reports whether adverse effects had been measured or not.

## 5. Conclusions

Although there were no valid lines of evidence of MT on improving dysfunction in patients with neck pain, this systematic review found moderate evidence of MT on improving pain in patients with neck pain compared with inactive therapies and limited evidence compared with traditional Chinese medicine due to few eligible studies. These are beneficial evidence of MT for neck pain. Assuming that MT is at least immediately effective and safe, it might be preliminarily recommended as a complementary and alternative treatment for patients with neck pain. But more high quality RCTs are urgently needed to confirm these results and continue to compare MT with other active therapies for neck pain.

## Figures and Tables

**Table 1:** Characteristics of included randomized controlled trials.

First authors, year, country	Pain duration	Sample size, mean age (year)	Duration weeks	Follow-up weeks	Main outcome assessments	Experimental group intervention*	Control group intervention*	Main conclusion (mean improvements on pain)
Irnich [17] 2001 Germany	42% >5 years	177 52	3	12	Pain VAS (0–100) Cervical mobility	Massage therapy (MT) (30 min/5 sessions)	(1) Acupuncture (AC) (2) Sham laser AC (30 min/5 sessions)	MT (12.70) < AC (25.30); MT (12.70) < sham laser AC (19.20)
Cen [18] 2003 USA	NR	31 49	6	6	Pain NPQ (0–100) ROM	Chinese traditional massage (CTM) (30 min/18 sessions)	(1) Exercise (EX) (20 min/day) (2) Standard care (SC)	CTM (19.22) > EX (7.58) CTM (19.22) > SC (−4.13)
Fryer [19] 2005 Australia	NR	37 23	1 day	—	PPT	Manual pressure release (MPR) (1 session)	Sham myofascial release (SMR) (1 session)	MPR (2.05) > SMR (−0.08)
Meseguer [20] 2006 Spain	NR	54 40	1 day	—	Pain VAS (0–10)	Classical strain/counterstrain technique (CST) Modified strain/counterstrain technique (MST) (1 session)	SC	CST = MST (2.60) CST (2.60) > SC (0.03)
Zaproudina [21] 2007 Finland	11.2 years	105 42	1 or 2	48	Pain VAS (0–100) NDI (0–100)	MT (30 min/5 sessions)	(1) Traditional bone setting (TBS) (90 min/5 sessions) (2) Physical therapy (PT) (45 min/5 sessions)	MT (21.20) < TBS (31.60) MT (21.20) > PT (17.20)
Blikstad [22] 2008 UK	4–12 weeks	45 24	1 day	—	Pain VAS (0–10) ROM	Myofascial band therapy (MBT) (1 session)	(1) Activator trigger point therapy (ATPT) (2) Sham ultrasound (SU) (1 session)	MBT < ATPT MBT = SU
Zuo [23] 2008 China	10.4 years	60 42	2	—	Pain VAS (0–10) NDI (0–50)	CTM (30 min/6 sessions)	Traction (TR) (20 min/14 sessions)	CTM (5.47) > TR (4.87)
Sherman [24] 2009 USA	7.6 years	64 47	10	16	NDI (0–50) CNFDS	MT (10 sessions)	SC	NDI: MT (5.50) > SC (2.20)
Jiang [25] 2010 China	—	60 <60	3	—	Pain VAS (0–10)	CTM (30 min/18 sessions)	Traditional Chinese medicine (TCM) (2/18 sessions)	CTM (3.40) > TCM (2.16)
Madson [26] 2010 USA	37.9 months	23 50	4	—	Pain VAS (0–100) NDI (0–50)	MT plus moist heat packs and EX (60 min/8–12 sessions)	Joint mobilization (JM) plus moist heat packs and EX (60 min/8–12 sessions)	MT (8.50) < JM (24.45)
Liu [27] 2011 China	31.6 months	90 42	2	—	Pain VAS (0–10) NDI (0–50) ROM	CTM (30 min/10 sessions)	(1) AC in abdomen (2) AC in neck and shoulder (30 min/10 sessions)	CTM (3.97) < AC1 (4.78) CTM (3.97) < AC2 (5.93)
Zhang [28] 2011 China	1–3 years	120 23	10 days	24	Pain VAS (0–10)	CTM (20 min/10 sessions)	TR (15 min/10 sessions)	CTM (5.56) > TR (3.85)
Lin [29] 2012 China	7.7 months	70 33	4	—	Pain VAS (0–10) ROM	CTM (12 sessions)	TCM (3/28 sessions)	CTM (4.17) > TCM (3.49)
Wang [30] 2012 China	1 week–5 years	66 38	2	—	Pain VAS (0–100)	CTM (20 min/6 sessions)	TR (20 min/6 sessions)	CTM (2.38) > TR (1.39)
Topolska [31] 2012 Poland	50% >11 years	60 63	10–15 days	—	Pain VAS (0–10) NDI (0–50) ROM	MT plus PT and kinesiotherapy (NR)	PT and kinesiotherapy (NR)	MT (1.40) < control (1.63)

VAS: visual analog scale; ROM: range of motion; NR: not reported; NPQ: Northwick park neck pain questionnaire; PPT: pressure pain threshold; NDI: neck disability index; CNFDS: Copenhagen neck functional disability scale.

*Intervention/dose: number of intervention times/number of sessions, number of Chinese herbal medicines every day/number of sessions.

**Table 2:** Studies excluded in full text screening.

Studies	Reason for exclusion
Chen et al. (2010) [32]	Intervention: multimodal including massage, mobilization, and manipulation
Fan (2010) [33]	Intervention: massage and manipulation
Fan et al. (2011) [34]	Intervention: massage and manipulation
Fu and Yuan (2001) [35]	Intervention: massage and manipulation
Huang (2010) [36]	Intervention: massage and Chinese herb
König et al. (2003) [37]	Duplicate publications as Irnich et al. (2001) [17]
Li and Fan (2001) [38]	Intervention: massage and manipulation
Lin et al. (2004) [39]	Intervention: multimodal including massage, mobilization, and manipulation
Lin et al. (2011) [40]	Duplicate publications as Lin et al. (2012) [29]
Li (2012) [41]	Intervention: massage and manipulation
Mai et al. (2010) [42]	Intervention: high-velocity and low-amplitude manipulation
Pan (2011) [43]	Intervention: multimodal including massage, mobilization, and manipulation
Qu and Wang (2012) [44]	Intervention: massage or manipulation
Sefton et al. (2011) [45]	Participants: healthy adults
Tan (2010) [46]	Outcome: Traditional Chinese Medicine Treatment Effect Rating Scale is employed; it is a composite of clinical symptoms, physical examination, and activities of daily life
Wang (2010) [47]	Intervention: massage and mobilization
Yang and Li (1991) [48]	Intervention: multimodal including massage, mobilization, and manipulation
Ylinen et al. (2007) [49]	Intervention: multimodal including mobilization, traditional massage, and passive stretching
Zhang et al. (2005) [50]	Outcome: Transcranial Cerebral Doppler and clinical symptoms (headache, vertigo, etc.)
Zhang et al. (2011) [51]	Duplicate publications as Zhang et al. (2011) [28]
Zhao (2011) [52]	Intervention: massage or manipulation
Zhang and Yu (2012) [53]	Outcome: Traditional Chinese Medicine Treatment Effect Rating Scale is employed; it is a composite of clinical symptoms, physical examination, and activities of daily life
Zheng and Xu (2011) [54]	Outcome: Traditional Chinese Medicine Treatment Effect Rating Scale is employed; it is a composite of clinical symptoms, physical examination, and activities of daily life

**Table 3:** PEDro scale of quality for included trials.

Study	Eligibility criteria	Random allocation	Concealed allocation	Similar at baseline	Subjects blinded	Therapists blinded	Assessors blinded	<15% dropouts	Intention- to-treat analysis	Between- group comparisons	Point measures and variability data	Total
Irnich et al. [17]	1	1	0	1	0	0	1	1	1	1	1	7
Cen et al. [18]	1	1	0	1	0	0	1	1	0	1	1	6
Fryer and Hodgson [19]	1	1	0	0	1	0	1	0	1	1	1	6
Meseguer et al. [20]	1	1	0	1	0	1	1	1	1	1	1	8
Zaproudina et al. [21]	1	1	1	1	1	1	1	1	0	1	1	9
Blikstad and Gemmell [22]	1	1	1	1	1	0	1	0	0	1	0	6
Zuo et al. [23]	1	1	0	1	0	0	1	1	1	1	1	7
Sherman et al. [24]	1	1	1	1	0	0	1	1	1	1	1	8
Jiang [25]	1	1	0	1	0	0	0	1	1	1	1	6
Madson et al. [26]	1	1	1	1	0	0	1	1	1	1	1	8
Liu [27]	1	1	1	1	0	0	0	1	1	1	1	7
Zhang et al. [28]	1	1	1	1	0	0	0	1	1	1	1	7
Lin et al. [29]	1	1	1	1	0	0	0	1	0	1	1	6
Wang et al. [30]	1	1	0	1	0	0	0	1	1	1	1	6
Topolska et al. [31]	1	1	0	0	0	0	0	1	1	1	1	5

0: did not meet the criteria; 1: met the criteria.
